# Patients’ Experiences of Telehealth-Based Nutrition Interventions for Polycystic Ovary Syndrome in China: Qualitative Descriptive Study

**DOI:** 10.2196/77709

**Published:** 2025-10-28

**Authors:** Hongyu Chen, Yuanyuan Wang, Fangzhi Xu, Zhicheng Huang, Wei Chen

**Affiliations:** 1 School of Nursing Chinese Academy of Medical Sciences & Peking Union Medical College Beijing China; 2 Department of Clinical Nutrition, Department of Health Medicine Key Laboratory of Diabetes FSMP, State Administration for Market Regulation Peking Union Medical College Hospital, Chinese Academy of Medical Sciences and Peking Union Medical College Beijing China; 3 Department of Intensive Care Unit The First Affiliated Hospital of Zhejiang Chinese Medical University (Zhejiang Provincial Hospital of Chinese Medicine) Hangzhou China

**Keywords:** polycystic ovary syndrome, telehealth, nutritional therapy, qualitative research, digital health

## Abstract

**Background:**

Telehealth-based nutrition care is increasingly used for polycystic ovary syndrome (PCOS); yet, little is known about women’s real-world experiences with PCOS-specific telenutrition in tertiary care settings. Understanding these experiences can guide patient-centered service design.

**Objective:**

We aim to explore women’s lived experiences, barriers, and preferences regarding telehealth-based nutrition interventions for PCOS and to derive actionable design implications.

**Methods:**

We conducted a qualitative descriptive study at a tertiary clinical nutrition center in Beijing, China. Purposive sampling recruited 12 adult women with PCOS who had engaged in telenutrition for at least 3 months. We conducted one-on-one, semistructured video interviews via WeChat (Tencent Holdings Limited; approximately 45-60 minutes each) between February and March 2025; interviews were audio-recorded, transcribed verbatim, and analyzed using the reflexive thematic analysis by Braun and Clarke. Trustworthiness was enhanced through dual-coding, audit trails, reflexive memos, and member checking.

**Results:**

Four participant-framed themes captured a tension between high acceptability and unmet needs. (1) “It fits my life”—convenience and access: flexible scheduling, reduced travel, and greater privacy lowered practical and emotional barriers and fostered a sense of continuity. (2) “One size doesn’t fit me”—frictions undermining engagement: standardized guidance did not reflect metabolic individuality (eg, insulin resistance or cycle-related symptoms) or daily routines; usability issues (glitches or nonintuitive logging) and limited communication bandwidth or timeliness impeded use. (3) “I’m not just a diet”—emotional and behavioral responses: timely, empathic feedback increased agency and accountability and supported adherence, whereas impersonal or delayed interactions left emotional needs unmet, highlighting the need for integrated mental health support. (4) “Make it smarter and more human”—participant recommendations: priorities included data-informed personalization (integration of laboratory and body-composition data and symptoms), integrated tracking and feedback loops, proactive check-ins with response-time standards, options for peer support, and cognitive behavioral therapy–informed microlessons.

**Conclusions:**

Telenutrition for PCOS is acceptable and convenient, but often underpersonalized and psychologically undersupported. Design implications include integrating individual metabolic data, embedding mental health screening and brief supports, instituting dietitian-initiated follow-ups, and improving usability and interactive feedback. Addressing the metabolic, reproductive, and psychological complexity of PCOS is essential for effective, scalable telehealth-based nutrition services.

## Introduction

Polycystic ovary syndrome (PCOS) is one of the most prevalent endocrine and metabolic disorders affecting women of reproductive age, with a global prevalence estimated between 5% and 18% [1]. It is characterized by a constellation of symptoms including menstrual irregularities, hyperandrogenism, insulin resistance, obesity, and infertility [2-5], significantly impacting patients’ physical and psychological well-being [6]. Among these, insulin resistance and associated metabolic disturbances constitute critical therapeutic targets [7], as they substantially elevate the risk for type 2 diabetes, cardiovascular diseases, and other metabolic complications [8,9].

Nutrition-based interventions have become foundational in managing PCOS due to their role in improving insulin sensitivity, hormonal balance, and overall metabolic health [10]. Clinical guidelines and consensus statements strongly advocate individualized dietary strategies as first-line treatment for PCOS [7,11], emphasizing the importance of personalized approaches tailored to patients’ unique metabolic profiles and lifestyle preferences [12,13]. However, despite strong recommendations, traditional face-to-face nutritional consultations are often hindered by barriers such as limited accessibility, inconvenience, inadequate follow-up, and insufficient personalization of dietary advice [14].

Recently, digital health technologies and telemedicine platforms have emerged as promising tools to improve access to nutrition care, offering remote monitoring, personalized dietary coaching, and real-time feedback—features that may enhance patient engagement and self-management in chronic diseases [15-18]. Telemedicine has demonstrated benefits in conditions such as diabetes and obesity, including improved continuity of care and better metabolic outcomes [19,20]. PCOS, however, presents a distinct set of clinical challenges. Beyond its reproductive manifestations, PCOS is often accompanied by insulin resistance, hyperandrogenism, and chronic low-grade inflammation, which intersect with emotional distress, disordered eating behaviors, and motivation difficulties [11,21]. These multidimensional and highly variable presentations demand individualized, dynamic nutritional interventions that consider both metabolic and psychological complexity. Yet, most existing digital nutrition interventions remain protocol-driven—for example, static meal plans or templated recommendations that are not iteratively adjusted to individual biomarkers; fasting insulin or the homeostatic model assessment of insulin resistance; and menstrual-cycle patterns, or cultural dietary habits—and insufficiently tailored, risking limited relevance and engagement for this unique population.

Beyond evidence from diabetes and obesity, PCOS-specific telehealth-based nutrition interventions have begun to emerge. Randomized trials show that app- or WeChat-based lifestyle programs can reduce weight and improve metabolic or androgenic markers with effects comparable to metformin in some outcomes [22-24]. Scoping and quality assessments indicate that PCOS apps are widely used for education, tracking, and behavior support, but the clinical quality and personalization are variable [25-27]. Despite these advances, the field lacks in-depth qualitative evidence on how women with PCOS actually experience telehealth-based nutrition care—particularly perceptions of personalization, usability, and emotional support—and how these experiences should inform design. Addressing this gap is essential to translate digital tools into patient-centered, clinically meaningful nutrition services for PCOS.

Understanding patient experiences is essential for the development of responsive and effective digital interventions [28]. Patient-centered approaches—incorporating user perspectives, preferences, and feedback—have been shown to increase the success of telehealth programs [29,30]. Therefore, exploring the experiences of women with PCOS using telehealth-based nutrition services is a necessary step to optimizing care delivery and informing more adaptive, personalized digital models [31].

Accordingly, this qualitative descriptive study aimed to provide an in-depth account of how women with PCOS experience telehealth-based nutrition care in routine practice and to derive concrete, patient-centered design implications for PCOS-specific digital nutrition services. To operationalize this aim, we addressed three research questions (RQs): (RQ1) How do women describe the benefits and challenges of telehealth-based nutrition in their everyday care? (RQ2) Which factors—particularly personalization, usability, communication patterns, and psychological support—act as barriers or facilitators of engagement and adherence? (RQ3) What specific features and workflows do patients prefer to better meet their metabolic and psychological needs?

## Methods

### Overview

This qualitative study used a descriptive design and followed the COREQ (Consolidated Criteria for Reporting Qualitative Research) guidelines to ensure methodological rigor, transparency, and thorough reporting (Multimedia Appendix 1) [32].

### Setting and Participant Recruitment

Participants were recruited from the Clinical Nutrition Department at Peking Union Medical College Hospital, a tertiary referral hospital in Beijing, China. The department primarily serves patients from Beijing and neighboring provinces in North China, with additional referrals from other regions nationwide. Purposive sampling was used to achieve a diverse range of participants in terms of age, occupation, PCOS severity, and duration of using platform-based telehealth nutrition services.

Women were eligible to participate if they met the following inclusion criteria: (1) aged 18 years or older; (2) diagnosed with PCOS based on the Rotterdam criteria [33]; (3) had engaged in telehealth-based nutrition counseling for ≥3 consecutive months; and (4) were able to communicate effectively in Mandarin and provide informed consent. We set a minimum exposure of ≥3 consecutive months to ensure adequate exposure to core service components (baseline dietetic assessment, iterative individualized feedback, and at least 2 follow-up cycles), allowing participants to reflect on sustained use beyond initial onboarding. Exclusion criteria included severe psychiatric illness, current pregnancy, or coexisting endocrine disorders. We excluded severe psychiatric illness to safeguard participant well-being, ensure capacity for informed participation in interviews, and avoid confounding of emotional experiences requiring specialized mental health management beyond the scope of nutrition services. Pregnancy was excluded because gestational physiology and obstetric care standards entail distinct nutritional requirements (eg, gestational weight-gain targets, micronutrient supplementation, and pregnancy-specific glycemic thresholds) and risk considerations that differ from routine PCOS management.

We identified eligible participants from clinical records from February to March 2025. Initial invitations to participate were conducted via telephone or WeChat messages [34]. Of the 18 patients invited, 12 agreed to participate. Reasons for declining participation included scheduling conflicts or privacy concerns.

Recruitment continued until thematic saturation was reached, indicated by no new themes emerging in the last 2 interviews [35]. Minimal information about the specific content of the interview was provided in advance to avoid prepared responses and promote spontaneity during interviews [36].

### Platform Context

The telehealth-based nutrition service was an institutionally operated (noncommercial) program delivered through a hospital-managed portal; it also supported patient-preferred communication via WeChat. We characterized the telehealth-based nutrition service as a hybrid model combining synchronous video consultations with dietitians and asynchronous in-app or WeChat messaging. Core functionalities included delivery and iterative adjustment of dietary plans, Q&A messaging, optional food-log and photo uploads, and patient-initiated upload of laboratory results and body composition assessments to inform personalization. Educational materials (brief articles and microvideos curated by the clinical team) were accessible within the platform. Personalization drew on available clinical data (eg, body composition; when available, indirect calorimetry measurements; fasting glucose or insulin or other metabolic markers shared by participants), reported symptoms and preferences (eg, menstrual-cycle regularity, hirsutism concerns, and satiety patterns), and lifestyle constraints (eg, work shifts). Nutrition plans were authored by licensed dietitians (not generated by automated algorithms), using behavior-change techniques (eg, goal setting, self-monitoring, problem-solving, and motivational interviewing) to support adherence.

### Intervention Description and Dose

At the minimum exposure of ≥3 consecutive months, participants experienced a structured sequence of components: (1) an initial baseline video consultation (comprehensive dietetic assessment; individualized nutrition plan aligned with PCOS guideline–consistent principles such as glycemic load management, adequate protein or fiber, and meal-timing strategies); (2) approximately monthly follow-up video consultations at ≈4-6–week intervals to review self-monitoring data (eg, food logs or photos), address barriers, and adjust the plan; and (3) ongoing asynchronous messaging during clinic hours for clarifications, brief check-ins, and tailored tips between visits. During month 1, onboarding emphasized assessment, goal setting, and first-plan implementation with early troubleshooting via messaging; by month 2-3, participants typically completed at least 2 feedback cycles with plan adjustments based on progress, participants’ preferences, and any new laboratory or body composition data. Optional modules (participant-driven) included uploading meal photos for feedback, accessing brief educational items, and sharing external laboratory reports for plan recalibration; use of these features varied by individual preference.

### Ethical Considerations

Ethical approval was granted by the Institutional Review Board of Peking Union Medical College Hospital (K-5204). All participants provided written informed consent, detailing confidentiality, voluntary participation, and withdrawal rights. Participants were explicitly informed about audio-recording and transcription processes, and all data were anonymized. As acknowledgment of their participation, instead of monetary compensation, participants received free clinical nutrition services, including personalized dietitian consultations, body composition assessments, and indirect calorimetry for metabolic measurements.

### Interview Guide and Data Collection

An initial interview guide was collaboratively developed based on a comprehensive literature review, expert consultation, and iterative pilot testing with 2 patients not included in the final study [37]. Interviews explored participant experiences with telehealth-based nutrition services, perceived advantages and barriers, personalization, and their recommendations for improvement. The complete semistructured interview guide is available in Multimedia Appendix 2.

Interviews were conducted by 2 researchers: HC (male, PhD in Nursing, clinical research nurse) and YW (female, MSc Nursing, clinical research nurse). Both interviewers had extensive experience in qualitative research and no prior relationships with the participants. Interviews were conducted individually via WeChat video call, lasting approximately 45-60 minutes, and took place between February and March 2025. Interviews were audio-recorded, transcribed verbatim by 2 independent professional transcriptionists, and double-checked by researchers for accuracy [38]. Detailed field notes were recorded during and immediately following each interview, capturing additional contextual and nonverbal information. No individuals other than the interviewer and participant were present during any interview.

### Data Analysis

We adopted the 6-step reflexive thematic analysis approach by Braun and Clarke [39] to guide the analysis process, which allowed flexible yet systematic identification of patterns within the data. The steps included (1) familiarization with the data, (2) generating initial codes, (3) searching for themes, (4) reviewing themes, (5) defining and naming themes, and (6) producing the report.

To enhance the transparency and dependability of analysis [40], 2 trained qualitative researchers (HC and YW) independently conducted line-by-line coding on 3 purposefully selected transcripts during the initial phase. These 3 transcripts were chosen to reflect participant diversity (age, occupation, and digital literacy) and richness of content, aiming to establish a preliminary coding framework that could accommodate heterogeneity across cases. Following independent coding, the coders compared and discussed discrepancies in regular meetings moderated by a third team member (FX), until a consensus-based initial codebook was developed [41].

The remaining transcripts were then coded using the evolving codebook. Both coders maintained analytic memos to track conceptual developments [42]. As coding progressed, codes were refined, collapsed, or expanded as needed. Emerging patterns were iteratively reviewed and grouped into candidate themes and subthemes during weekly discussions [43]. Thematic saturation was confirmed when no new themes emerged during the coding of the final transcripts (participants 11 and 12). NVivo 12 software (Lumivero) was used for coding management [44].

### Trustworthiness and Rigor

To enhance trustworthiness, we applied the criteria by Lincoln and Guba [45] in an integrated paragraph rather than a list: credibility was supported through dual-coding, regular team debriefs, and member checking of preliminary interpretations [46]; dependability through detailed audit trails documenting coding decisions and code evolution [40], independently reviewed by a team member not involved in initial coding (ZH); transferability via thick description of the setting, sampling strategy, participant characteristics, and thematic content [47]; and confirmability by maintaining reflexive memos and an auditable analytic record, with dual-coder triangulation minimizing interpretive bias [32].

## Results

### Description of Study Participants

Twelve women diagnosed with PCOS participated; insulin resistance was present in 7/12 (58%) participants per the threshold. All participants were recruited from the Clinical Nutrition Department at Peking Union Medical College Hospital. The mean age was 28.7 (SD 4.7, range 22-38) years. Participants varied in educational background and employment status, with most holding at least a bachelor’s degree. Seven participants were employed full-time, 3 were postgraduate students, and 2 were full-time homemakers. Marital status: unmarried, 6/12 (50%); married, 4/12 (33%); and divorced or widowed, 2/12 (17%). BMI categories: 18.5-23.9, 2/12 (17%); 24.0-27.9, 3/12 (25%); and ≥28.0, 7/12 (58%). Rotterdam phenotypes [48]: A, 7/12 (58%); B, 2/12 (17%); C, 2/12 (17%); and D, 1/12 (8%). The most commonly reported presenting problems included menstrual irregularity and clinical hyperandrogenism (eg, hirsutism or acne), with weight concerns and mood symptoms variably noted.

Participants reported a mean duration since their PCOS diagnosis of 4.2 (SD 2.7, range 1-10) years and had been using the telehealth-based nutrition intervention for an average of 5.6 (SD 2.9, range 3-12) months. All participants had previously received varying degrees of traditional nutritional counseling before transitioning to telehealth-based nutrition services. Demographic characteristics are presented in Table 1, and clinical characteristics in Table 2.

**Table 1:** Demographic characteristics of participants.

Characteristics			Participants (N=12), n (%)
**Age (years)**			
	18-25	3 (25)	
	26-30	5 (42)	
	31-35	3 (25)	
	36-40	1 (8)	
**Ethnicity**			
	Han	6 (50)	
	Hui	3 (25)	
	Manchu	2 (17)	
	Mongol	1 (8)	
**Marital status**			
	Unmarried	6 (50)	
	Married	4 (33)	
	Divorced or widowed	2 (17)	
**Education level**			
	Bachelor’s degree	6 (50)	
	Master’s degree	5 (42)	
	Doctoral degree	1 (8)	
**Occupation**			
	Full-time employee	7 (58)	
	Postgraduate student	3 (25)	
	Homemaker	2 (17)	
**Duration of PCOS^a^ diagnosis (years)**			
	1-3	5 (42)	
	4-6	4 (33)	
	7-10	3 (25)	
**Duration of telehealth-based nutrition (months)**			
	3-5	4 (33)	
	6-8	5 (42)	
	≥9	3 (25)	

^a^PCOS: polycystic ovary syndrome.

**Table 2:** Clinical characteristics of participants.

Characteristics			Participants (N=12), n (%)
**BMI (kg/m^2^)**			
	18.5-23.9	2 (17)	
	24.0-27.9	3 (25)	
	≥28.0	7 (58)	
**Rotterdam phenotype**			
	A (HA^a^+OA^b^+PCO^c^)	7 (58)	
	B (HA+OA)	2 (17)	
	C (HA+PCO)	2 (17)	
	D (OA+PCO)	1 (8)	
**HOMA-IR^d^**			
	≤1.66	5 (42)	
	>1.66	7 (58)	

^a^HA: hyperandrogenism.

^b^OA: oligo or anovulation.

^c^PCO: polycystic ovaries.

^d^Homeostatic model assessment of insulin resistance=(fasting glucose [mmol/L]×fasting insulin [μU/mL])/22.5; institutional threshold for insulin resistance: homeostatic model assessment of insulin resistance>1.66.

### Theme 1: “It Fits My Life”—Convenience and Access From Participants’ Perspectives

#### Overview

Participants framed telehealth-based nutrition as something that finally “fit” into busy, geographically dispersed lives; convenience was not merely logistical but a precondition for uptake and continuity.

#### Subtheme 1.1: “I Can See My Dietitian When It Suits Me”—Flexibility in Scheduling and Location

A key benefit frequently emphasized was the increased flexibility offered by telehealth services, which participants described as especially valuable due to their busy professional or personal schedules. The flexibility to arrange consultations outside typical hospital hours helped many integrate nutritional counseling seamlessly into their daily lives.

I have a demanding job and often work overtime, so visiting the hospital during regular working hours was almost impossible for me. With telehealth-based nutrition, I can schedule consultations in the evening or during weekends, making it much more practical to get regular guidance.

Participant 2

Another participant similarly stated:

My studies and research occupy most of my weekdays, and I previously had to skip nutritional consultations frequently. Telehealth-based nutrition has dramatically reduced this issue by enabling sessions from my dormitory after class. It fits perfectly around my tight schedule.

Participant 3

Participants living outside urban areas also expressed particular appreciation for the convenience:

I used to travel two hours each way to the hospital, which was exhausting. Telehealth consultations have entirely removed the travel burden and allowed me to consult my nutritionist regularly without the stress and expense of long journeys.

Participant 9

#### Subtheme 1.2: “I’m More Likely to Reach Out”—Lowered Practical and Emotional Barriers

Participants linked convenience to a lower threshold for seeking help, emphasizing not only time or travel savings but also reduced embarrassment and greater perceived privacy.

Going to the hospital frequently felt overwhelming because of the waiting times and crowds, especially after a long workday. Now, logging into my consultation at home eliminates this stress, making me more likely to seek guidance consistently.

Participant 5

In our context—an urban, high-volume tertiary referral hospital in China—outpatient clinics often experience heavy demand and queuing, which several participants referenced when contrasting in-person visits with telehealth. Others mentioned that telehealth reduced the emotional and social discomfort of face-to-face interactions in public settings:

I was often embarrassed to discuss personal issues like weight struggles and emotional eating in a busy outpatient environment. Online consultations feel more private and less judgmental, allowing me to speak openly and get more authentic help.

Participant 8

A few participants emphasized how the ease of app-based access motivated them to seek help proactively rather than delaying care:

Before telehealth-based nutrition, I often delayed seeking dietary advice until my symptoms worsened. Now, because consultations are just a click away, I am much more proactive about asking questions and managing my symptoms early.

Participant 10

#### Subtheme 1.3: “It Feels Continuous, Not Episodic”—Continuity of Care and Support

Participants described telehealth as transforming care from sporadic, visit-based encounters to a sense of ongoing connection, which they perceived as essential for adherence.

My nutritionist previously gave me dietary plans that were difficult to implement without ongoing support. Now, the online platform allows regular check-ins and quick feedback, which helps me sustain positive dietary habits and remain accountable.

Participant 1

Several participants appreciated the real-time, 2-way features of digital (app-based) nutrition platforms, noting these significantly improved their adherence to dietary interventions:

Having constant access to nutritional guidance through online chats or short follow-up calls helps me quickly address questions about food choices or symptoms. It feels like continuous support rather than intermittent visits, which is incredibly reassuring and encouraging.

Participant 6

Furthermore, participants highlighted how telehealth created a sense of being closely supported through personalized feedback loops:

The personalized follow-up I get from telehealth-based nutrition—such as reminders, motivational messages, or brief check-ins from my nutritionist—makes me feel cared for and keeps me focused on my dietary goals. I never experienced this degree of continuity with face-to-face sessions.

Participant 7

Finally, continuity of care was also reflected in participants’ experiences of better communication with their care providers:

Telehealth allowed my nutritionist to closely monitor my progress, regularly adjusting my diet plans based on my actual feedback. This immediate responsiveness made a huge difference in managing my insulin resistance symptoms effectively.

Participant 12

### Theme 2: “One Size Doesn’t Fit Me”—Frictions That Undermined Engagement

#### Overview

Despite overall acceptance, participants emphasized that generic guidance, platform frictions, and communication bandwidth issues impeded engagement and adherence.

#### Subtheme 2.1: Generic Guidance Versus Individualized Needs

Participants frequently expressed dissatisfaction with the generic nature of dietary advice provided by telehealth-based nutrition services. Many felt that standardized dietary recommendations did not adequately reflect their individual metabolic concerns or personal circumstances related to insulin resistance and PCOS.

One participant explained:

The advice given often felt very general, like something you could find online easily. It rarely considered my specific issues, like insulin spikes or cravings linked to hormonal fluctuations. I needed more tailored and personalized support.

Participant 4

Another participant added similar frustrations:

My situation is unique—I have specific dietary restrictions and particular insulin-resistance problems. But the telehealth-based nutrition platform often suggested similar meal plans to everyone. It didn’t feel adapted to my actual health needs and daily routine.

Participant 10

Participants clearly indicated that generic guidance sometimes led to confusion or dissatisfaction, reducing their motivation to adhere:

I found myself repeatedly needing more precise dietary guidance based on my blood test results or metabolic parameters. When I didn't get that from the tele-platform, it became hard to trust the recommendations fully or follow through consistently.

Participant 12

#### Subtheme 2.2: Technology and Usability as “Hidden Workload”

Participants portrayed technical instability and confusing interfaces as a hidden workload that diverted energy away from behavior change.

A participant clearly articulated the frustration of dealing with repeated technical glitches:

The video calls often froze or disconnected midway through a consultation. I lost a lot of valuable time trying to reconnect. Sometimes, sessions became so fragmented and frustrating that it discouraged me from using the service altogether.

Participant 1

Participants also experienced difficulties related to the complexity and unintuitive nature of platform design:

I’m not tech-savvy. The app had many functions that were difficult to find or understand. Trying to upload food logs or health data was frustrating, and sometimes I ended up not uploading them at all because of the confusing interface.

Participant 5

Furthermore, inadequate technical assistance amplified these problems:

Whenever I faced technical issues, the support from the platform was slow or insufficient. One time I waited almost a week to get a response about an issue, during which my dietary tracking was completely interrupted.

Participant 8

#### Subtheme 2.3: Communication Bandwidth and Timeliness

Participants associated limited real-time responsiveness and the reduced richness of app-based communication with unmet informational and emotional needs.

One participant shared her experience of limited communication effectiveness:

Online consultations felt rushed compared to face-to-face meetings. I often ended sessions feeling that many of my concerns weren't fully addressed because we ran out of time or couldn't communicate effectively through video.

Participant 3

Others reported difficulties in clearly conveying their concerns due to reduced nonverbal communication and a lack of direct personal interaction:

Communicating complex nutritional or emotional concerns is challenging online. Sometimes I felt my nutritionist missed important details about my emotional state or struggles because we couldn't communicate effectively through a screen.

Participant 6

Additionally, participants mentioned delayed or generic feedback from health providers, which undermined the potential effectiveness of telehealth:

Sometimes I waited days for responses from my nutritionist on the platform. By then, I had already struggled with certain dietary decisions. I wish there were more immediate or interactive communication methods available.

Participant 7

One participant summarized how these communication limitations impacted her overall motivation:

Limited and delayed communication often made me feel isolated in managing my condition. Effective dietary management requires ongoing dialogue and support, and without that, my motivation definitely declined over time.

Participant 11

### Theme 3: “I’m Not Just a Diet”—Emotional and Behavioral Responses

#### Overview

Telehealth afforded agency and accountability for many participants, yet the absence of explicit psychological support sometimes left emotional needs unmet.

#### Subtheme 3.1: Empowerment and Agency

Several participants emphasized how telehealth-based nutrition interventions fostered a greater sense of personal empowerment and control in managing their dietary habits and PCOS-related symptoms. Regular interaction and immediate access to nutritional advice strengthened their self-management capabilities.

One participant expressed clear satisfaction regarding enhanced self-control:

Previously, I felt helpless because I didn’t have continuous access to dietary guidance. Now, I can quickly get feedback online when I face uncertainties, making me feel much more in control of my dietary decisions.

Participant 3

Another highlighted that regular monitoring improved her sense of accountability and effectiveness in self-management:

Having constant reminders and feedback through the platform really helps. I now feel responsible and accountable for my choices, which motivates me to adhere more closely to nutritional advice and monitor my health regularly.

Participant 9

Participants also described emotional confidence derived from regular positive reinforcement:

The regular affirmations from my online nutritionist—such as praising small dietary achievements—boosted my confidence significantly. These emotional boosts helped me maintain healthy eating behaviors much more consistently.

Participant 10

Across accounts, participants explicitly linked feeling “more in control” to concrete behaviors—more consistent food logging, meal planning, and follow-through—which they perceived as better adherence to dietetic recommendations. Conceptually, this links self-efficacy to adherence in a manner consistent with established behavior-change theories—namely the Social Cognitive Theory by Bandura [49] and the Capability–Opportunity–Motivation–Behavior model [50] —making downstream clinical improvement (eg, metabolic control) plausible.

#### Subtheme 3.2: Emotional Friction From Impersonal Interactions

Despite some positive emotional outcomes, participants also reported notable emotional distress and frustrations stemming from impersonal interactions or a lack of sufficient personalized emotional support within telehealth-based nutrition consultations.

Participants highlighted feelings of isolation and impersonal engagement:

Although convenient, online consultations can feel cold and impersonal. At times I felt emotionally disconnected because it’s harder to form a supportive relationship with someone through a screen compared to face-to-face.

Participant 2

Another participant echoed similar concerns, specifically pointing out the need for more empathic communication:

My nutritionist often focused heavily on what I should eat and neglected emotional issues like stress or anxiety related to eating. This felt emotionally isolating, as managing my condition is emotionally exhausting, not just physically.

Participant 8

The limited emotional depth of interactions also affected participants’ motivation:

The inability to truly express or discuss emotional struggles online felt limiting. Without deeper emotional support, maintaining dietary habits sometimes felt meaningless or exhausting.

Participant 5

#### Subtheme 3.3: Call for Integrated Psychological Support

A consistent and prominent need identified among participants was the desire for comprehensive psychological support integrated within telehealth-based nutrition interventions. Participants emphasized that effective dietary management required simultaneous psychological support, particularly given the emotional and psychological burden of managing PCOS and insulin resistance.

Participants openly advocated for integrated psychological support:

Managing PCOS isn't just about dietary advice. I face emotional eating, anxiety, and stress. I really wished my telehealth-based nutrition platform provided integrated psychological support—just dietary guidelines alone aren’t enough.

Participant 6

Another participant reinforced this perspective, emphasizing the importance of holistic management:

Nutrition interventions should be combined with psychological support. Emotional stress strongly affects my eating habits and symptoms, but these emotional aspects are rarely addressed sufficiently by nutritionists online.

Participant 7

Participants also expressed clear expectations for professional psychological interventions integrated into nutritional counseling:

Adding psychological support, such as online counseling sessions or group discussions focusing on emotional coping strategies, could really enhance the effectiveness of telehealth-based nutrition. Currently, I feel there's a significant gap in emotional management.

Participant 12

Lastly, some participants discussed proactive emotional strategies they attempted due to the lack of integrated support:

Without built-in psychological support, I often had to seek emotional advice elsewhere, making my management fragmented. If emotional support were integrated within the nutritional counseling platform, it would greatly streamline my care and enhance adherence.

Participant 11

### Theme 4: “Make It Smarter and More Human”—Participant Recommendations to Improve Telehealth-Based Nutrition

#### Overview

Participants’ suggestions converged on 2 design imperatives: stronger data-informed personalization and more human, proactive support.

#### Subtheme 4.1: Data-Informed Personalization (Laboratory Data, Body Composition, and Symptoms)

Participants emphasized the urgent need for nutritional advice tailored explicitly to their individual metabolic profiles, reflecting their unique hormonal and insulin-resistance status. They suggested integrating metabolic data such as blood glucose, hormone levels, and body composition into dietary planning to make interventions genuinely personalized.

One participant explained the necessity of personalized data-driven dietary advice:

Generic meal plans don't work for my PCOS. I wish my nutritionist could regularly check my actual blood tests or insulin response data, and then tailor my dietary recommendations accordingly. This could make a huge difference in managing my symptoms.

Participant 1

Another participant similarly emphasized integrating individual metabolic monitoring:

I hope future platforms will integrate data like glucose monitoring, hormone tests, or metabolic rates directly into dietary plans. Personalized recommendations based on such data would significantly improve my ability to follow and trust the guidance provided.

Participant 3

A participant clearly articulated how tailored advice based on metabolic metrics could enhance trust and motivation:

If dietary plans were clearly linked to my actual metabolic data, I would feel much more confident about following them. Generic advice often leaves me doubtful and confused, but data-driven guidance feels trustworthy and actionable.

Participant 6

#### Subtheme 4.2: Integrated Tracking and Feedback Loops

Participants frequently recommended adding comprehensive health-tracking tools within the telehealth-based nutrition platforms, such as diet and exercise logs, automated symptom tracking, and integrated feedback systems to provide more holistic support.

One participant explained her vision of an ideal platform:

An ideal telehealth-based nutrition platform would let me track meals, exercise, sleep, and even emotional states, all in one place. A comprehensive picture could then help my nutritionist give better, more holistic advice.

Participant 5

Another participant highlighted how integrated tracking would enhance self-management capabilities:

I would love a feature where I could log my daily dietary intake, physical activity, symptoms, and even emotional status. Automated weekly or monthly reports summarizing these data points could greatly assist me and my nutritionist in monitoring progress more effectively.

Participant 10

Furthermore, participants suggested automated alerts and feedback based on their tracking data:

If the platform could automatically identify patterns—such as a connection between eating certain foods and worsening symptoms—and alert me immediately, that would greatly improve my dietary management.

Participant 4

#### Subtheme 4.3: Proactive, 2-Way Professional and Peer Support

Participants preferred care that “reaches out,” not just “responds,” including brief check-ins, rapid messaging, and peer community features.

A participant described her expectations regarding more proactive support:

Nutritionists could be more proactive in initiating check-ins or motivational interactions. Currently, I mostly initiate contact myself. Regular proactive check-ins from nutritionists would significantly enhance my motivation and adherence.

Participant 2

Another participant underscored the value of prompt, 2-way in-app feedback:

Quick and interactive feedback from nutritionists is crucial. If I could receive immediate advice on daily dietary choices or emotional eating episodes through instant messaging within the platform, that would profoundly enhance my experience and outcomes.

Participant 8

Participants also suggested 2-way community support features within digital (app-based) platforms:

In addition to interactions with nutritionists, adding features like community groups, forums, or peer support chats within the telehealth-based nutrition service could provide additional motivation, emotional support, and practical tips.

Participant 7

Lastly, participants highlighted the benefits of regular feedback loops designed to sustain engagement and motivation:

If nutritionists regularly provided summaries of my progress or celebrated my dietary successes through short videos or personalized messages, it would keep me motivated and positively engaged long-term.

Participant 9

## Discussion

### Principal Findings

This qualitative descriptive study examined how women with PCOS experience a hybrid, hospital-operated telehealth-based nutrition program in China; interviews with 12 participants (≥3 months’ engagement) yielded 4 themes indicating high convenience and acceptability but gaps in personalization, usability, and psychological or communication support. For RQ1: theme 1, “it fits my life,” describes how convenience (flexible scheduling, reduced travel, and greater privacy in a high-volume tertiary outpatient context) lowered practical and emotional barriers to help-seeking; theme 2, “one size doesn’t fit me,” captures how generic guidance, platform or usability frictions, and slow or impersonal responses impeded engagement. For RQ2: theme 3, “I’m not just a diet,” shows that timely, empathic feedback and continuity bolstered self-efficacy and translated into better adherence, whereas underpersonalization and delayed communication undermined adherence. For RQ3: theme 4, “make it smarter and more human,” reflects priorities for data-informed tailoring (easy laboratory or body composition upload with iterative plan adjustment), embedded psychological supports (brief screening, stepped referrals, and cognitive behavioral therapy–informed microsupports), and proactive workflows (response-time standards, scheduled check-ins, and photo-based meal feedback). Household or partner dynamics also shaped meal planning and follow-through. Collectively, findings indicate that PCOS telehealth–based nutrition is acceptable but underpersonalized and psychologically undersupported, specifying actionable targets for service refinement (data-driven personalization, embedded mental health supports, and proactive communication norms).

These results are congruent with prior telehealth literature showing time-saving and access benefits for people with chronic conditions [51], including diabetes or obesity contexts where remote dietary counseling improves access and adherence [52,53].

Our contribution extends this evidence in 3 PCOS-specific ways. First, despite evidence for the effectiveness of medical nutrition therapy—individualized nutrition assessment and counseling delivered by credentialed dietitians—in managing insulin resistance and hyperandrogenism [54], participants reported that real-world telehealth-based nutrition often failed to reflect metabolic individuality (eg, insulin sensitivity, basal metabolic rate, and hormonal status), revealing a gap between best practice and digital delivery [55].

Second, while emotional eating and psychological distress are common in PCOS [56,57], participants described that digital encounters rarely addressed coping and motivation, underscoring the need for embedded psychological supports within nutrition care. Third, although 2-way, app-based capabilities such as wearables, real-time feedback, and clinician messaging can enhance engagement and outcomes in related populations [58], these features were perceived as underused in PCOS-specific nutrition services.

Taken together, our data refine the design target for PCOS telehealth–based nutrition beyond “generic chronic metabolic management.” Effective remote care must address the metabolic-reproductive-psychological triad unique to PCOS by (1) incorporating phenotype or cycle information into meal timing and macronutrient targets, (2) linking dietary adjustments to hyperandrogenic symptoms and weight-stigma experiences, and (3) embedding brief, stepped psychological supports alongside data-driven personalization. This PCOS-specific profile explains why standard diabetes-style, symptom-agnostic protocols are frequently experienced as insufficient in this population.

### Implications for Clinical Practice and Platform Design

The insights gathered from this study yield several important implications for both clinical nutrition practice and the future development of telehealth-based nutrition platforms, especially in the management of PCOS with insulin resistance.

First, clinicians and platform designers must acknowledge that standardized dietary guidance is insufficient for managing the heterogeneous and hormonally complex nature of PCOS [59]. Participants expressed a clear need for nutrition interventions that account for individual variations in insulin sensitivity, androgen levels, and body composition. To meet this need, telehealth platforms should incorporate mechanisms for collecting, interpreting, and integrating personalized clinical data, such as basal metabolic rate, glucose trends, and hormonal profiles, into individualized dietary recommendations. Practically, platforms can provide structured “laboratory upload” workflows (eg, fasting glucose or insulin and lipid profile) and auto-populate a clinician dashboard that visualizes trends and flags potential insulin-resistance risks for review; meal-photo feedback can be coupled with top-3 actionable changes (eg, protein swap, fiber add-on, and glycemic-load reduction) rather than generic tips.

Second, this study highlights the importance of emotional and behavioral integration into dietary counseling. Many patients identified emotional eating, body dissatisfaction, and psychological stress as major barriers to adherence, yet reported these issues were rarely acknowledged during remote consultations. Thus, nutritional care models for PCOS should be embedded within multidisciplinary frameworks (eg, collaborative care models that pair dietitians with psychologists or mental health clinicians—and, where relevant, endocrinologists—using shared care plans, brief joint sessions or covisits, case-conference reviews, and warm handoffs within the same episode of care) that include behavioral therapy, mental health support, and motivational interviewing. This integrative approach would not only address root causes of nonadherence but also improve patient engagement and satisfaction [60]. Platforms may consider optional family-inclusive features (eg, partner-facing summaries, shared meal plans, and brief joint check-ins) to leverage social support while respecting patient autonomy. Concretely, platforms should (1) embed brief validated mental health screeners at onboarding and periodically (eg, every 8-12 weeks)—such as the 9-item Patient Health Questionnaire [61] for depressive symptoms and the 7-item Generalized Anxiety Disorder scale [62] for anxiety—displaying results only to clinicians; (2) implement stepped-care triage rules (eg, 9-item Patient Health Questionnaire ≥10 triggers in-app psychoeducation plus a warm handoff to psychology; any suicidal ideation response prompts an immediate safety workflow and urgent referral per local protocol); (3) offer cognitive behavioral therapy–informed microlessons (eg, thought-reframing, urge-surfing, and coping-planning) and 3-5–minute guided breathing or grounding audios that can be sent as “first aid” when users report cravings or stress; and (4) provide optional referrals to mental health professionals and, where available, group-based skills sessions integrated into the platform calendar as well as scheduled dietitian-psychologist cosessions for patients with elevated screening scores.

Third, the usability and interactivity of digital platforms must be improved to reduce participation barriers and enhance ongoing engagement [63,64]. Several participants described the interface of existing telehealth-based nutrition systems as technically burdensome, with fragmented communication and poor data visualization. To remedy this, developers should adopt user-centered design principles that prioritize simplicity, personalization, and real-time feedback. Features such as artificial intelligence–assisted food logging, automated reminders, and visual dashboards linked to biometric data could substantially enhance the user experience. Additionally, platforms should define communication “service-level” standards (eg, dietitian responses to asynchronous messages within 1 business day and proactive 2-4–week check-ins by default) and provide templated progress summaries after each visit (goals set, barriers noted, and next steps), which participants in this study explicitly sought.

Moreover, patients emphasized the need for longitudinal and dynamic support, rather than 1-time consultations [65]. Clinicians should consider transitioning from episodic care to continuous care models, wherein patients receive routine follow-ups, adaptive feedback, and timely adjustments to their dietary plans based on evolving clinical data and patient-reported outcomes.

Lastly, platforms should consider facilitating peer support or group-based features, allowing patients to share experiences and strategies [66]. Peer interaction may reduce isolation, normalize challenges, and reinforce behavior change—a particularly valuable strategy for populations coping with chronic conditions that carry emotional stigma.

Collectively, these findings underscore the urgent need for a paradigm shift in telehealth-based nutrition care for PCOS—from passive, protocol-driven models to proactive, personalized, and emotionally intelligent systems that reflect the lived realities of the patients they serve [67,68].

### Limitations

Small, single-center samples (n=12) from urban China limits transferability. Contextual factors—tertiary-care referral patterns, high outpatient volumes, and routine use of WeChat for health communication—may have shaped perceived convenience and interaction norms; generalizability to Western primary care or lower-resource settings is uncertain without cultural or system adaptation. This study captured patient perspectives only (no dietitians, endocrinologists, or developers), and lacked objective usage analytics (eg, logins or task completion) to complement narratives. Interviewer-participant gender mismatch (1 male interviewer and female participants) could have influenced disclosure despite mitigation (standardized, nonjudgmental prompts and private one-to-one interviews). As a qualitative study, findings do not establish causality; mixed methods and intervention trials should test whether specific platform features improve clinical outcomes.

Overall, this study identifies actionable targets for PCOS telehealth–based nutrition—data-driven personalization, embedded mental health supports, and proactive communication workflows—despite the above constraints.

### Conclusions

This qualitative study sheds light on the lived experiences, barriers, and expectations of women with PCOS in accessing platform-based telehealth nutrition services. While participants appreciated the convenience and accessibility of digital interventions, they emphasized that current telehealth-based nutrition models often fall short in providing personalized, metabolically tailored, and emotionally supportive care.

The findings underscore an urgent need to move beyond generic dietary guidance toward integrated, adaptive, and user-centered approaches. Clinicians and developers must work collaboratively to build platforms that incorporate individualized metabolic data, behavioral and emotional support, and seamless user interfaces. Only through such innovations can telehealth-based nutrition interventions truly meet the complex and evolving needs of women managing PCOS.

Future research should explore multi-stakeholder perspectives, evaluate effectiveness through intervention trials, and leverage digital tools to create scalable, evidence-based models of precision nutrition care for chronic metabolic conditions.

## Appendix

Multimedia Appendix 1 COREQ: 32-item checklist.

Multimedia Appendix 2 Interview guide.

## Abbreviations

COREQ: Consolidated Criteria for Reporting Qualitative Research
PCOS: polycystic ovary syndrome
RQ: research question

## Data Availability

The datasets generated during or analyzed during this study are available from the corresponding author on reasonable request.
