# Supplementary material for: Patients’ Experiences of Telehealth-Based Nutrition Interventions for Polycystic Ovary Syndrome in China: Qualitative Descriptive Study
Source: J Med Internet Res. 2025 Oct 28;27:e77709. doi: 10.2196/77709 (PMC12605266; doi:10.2196/77709)
Supplement: Multimedia Appendix 2 [file jmir_v27i1e77709_app2.docx]

| Q1: What motivated you to use telehealth-based nutrition services?  Q2: Could you describe your experiences and impressions using the telehealth platform for nutritional support?  Q3: What difficulties or frustrations have you encountered during your telehealth-based nutrition counseling?  Q4: How do you perceive the adequacy of the telehealth-based nutrition interventions in addressing your metabolic concerns, especially insulin resistance?  Q5: How could telehealth-based nutrition services better meet your individual nutritional needs?  Q6: What additional support or features would you like integrated into future telehealth-based nutrition platforms?  Q7: How do you find the technical usability of the current telehealth-based nutrition platform?  Q8: Have you encountered any issues related to technology access or functionality? |
| --- |
